# Identification of fungus-growing termite-associated halogenated-PKS maduralactomycin a as a potential inhibitor of MurF protein of multidrug-resistant *Acinetobacter baumannii*

**DOI:** 10.3389/fmolb.2023.1183073

**Published:** 2023-04-21

**Authors:** Muhammad Shoaib, Iram Shehzadi, Muhammad Umair Asif, Yulong Shen, Jinfeng Ni

**Affiliations:** ^1^ State Key Laboratory of Microbial Technology, Microbial Technology Institute, Shandong University, Qingdao, Shandong, China; ^2^ Basic Health Unit, Toba TekSingh, Pakistan; ^3^ Basic Health Unit, Faisalabad, Pakistan

**Keywords:** MurF protein, natural products screening, and molecular dynamics, *Acinetobacter baumannii*, fungal-associated termite

## Abstract

Multidrug-resistant *Acinetobacter baumannii* infections have become a major public health concern globally. Inhibition of its essential MurF protein has been proposed as a potential target for broad-spectrum drugs. This study aimed to evaluate the potential of a novel ecological niche of 374 fungus-growing termite associated Natural Products (NPs). The molecular docking and computational pharmacokinetics screened four compounds, i.e., Termstrin B, Fridamycin A, Maduralactomycin A, and Natalenamide C, as potential compounds that have higher binding affinities and favourable protein-ligand interactions. The compound Maduralactomycin A induced more stability based on its lowest average RMSD value (2.31 Å) and low standard deviation (0.35) supported by the consistent flexibility and β-factor during the protein’s time-dependent motion. While hydrogen bond analysis indicated that Termstrin B has formed the strongest intra-protein interaction, solvent accessibility was in good agreement with Maduralactomycin A compactness. Maduralactomycin A has the strongest binding energy among all the compounds (−348.48 kcal/mol) followed by Termstrin B (−321.19 kcal/mol). Since these findings suggest Maduralactomycin A and Termstrin B as promising candidates for inhibition of MurF protein, the favourable binding energies of Maduralactomycin A make it a more important compound to warrant further investigation. However, experimental validation using animal models and clinical trials is recommended before reaching any final conclusions.

## Introduction

*Acinetobacter baumannii*, a Gram-negative bacterium, is one of the top six ESKAPE pathogens responsible for multidrug-resistant (MDR) hospital-acquired infections (Cho et al., 2018; Ma et al., 2020; Motbainor et al., 2020). Pneumonia, bacteremia, urinary tract infections, meningitis, and wound infections are frequently caused by *A. baumannii*, especially in burn patients (Correa et al., 2018). Although the bacteria developed inherent resistance to a variety of antibiotics, further acquired resistance has emerged during the past three decades (Presta et al., 2017). *A. baumannii* resistance to imipenem grew to 85% in certain locations, posing serious risks to the public’s health. According to studies, hospital death rates are high, ranging from 50% to 64%, and the majority of patients pass away within 48 h of being admitted (A Rahman et al., 2017). MDR *A. baumannii* epidemics are widespread, and cases have been documented worldwide, including in Iraq, India, Spain, Germany, Brazil, Turkey, the United States, Japan, Iran, and the UK (Skariyachan et al., 2019). Reports suggested that *A. baumannii* is the most severe pathogen which shows resistance against diverse classes of antibiotics. Among the various pathways found in MDR *A. baumannii*, the peptidoglycan biosynthetic pathway is the most important for the survival of the bacteria (Moubareck and Halat, 2020).

Peptidoglycan (PG) an essential component of the bacterial cell wall, is a target of choice for several antibacterial drugs (Gu et al., 2004). Bacterial peptidoglycan is made up of biological macromolecules that cover cell membranes and components of the cell walls. This bacterial cell wall is responsible for shape and firmness as well as protecting cells from bursts because of variations in osmotic pressure. In general, peptidoglycan is the chief constituent of the outer membrane of the majority of eubacteria cell walls, including A. *baumannii (* *Saxena et al., 2018* *).* The Mur family of enzymes catalyze intracellular ATP-driven reactions and have similar reaction mechanisms and three-dimensional (3D) architectures (Barreteau et al., 2008). The MurF enzyme is responsible for the incorporation of a d-alanyl d-alanyl moiety during peptidoglycan synthesis and has been identified as a promising target for a variety of reasons (Smith, 2006; Barreteau et al., 2008; Hrast et al., 2014). Primarily, MurF is essential for the bacteria to survive, secondly, as MurF is highly conserved in both Gram-positive and Gram-negative bacteria, there may be potential to develop broad-spectrum therapeutic agents (El Zoeiby et al., 2003). Thirdly, MurF function seems to be indispensable for development of resistance against the lactam antibiotics (Sobral et al., 2003). Lastly, disrupting the MurF function impairs bacteria’s capacity for replication (El Zoeiby et al., 2003). Antibiotic-inactivating enzymes decreased bacterial entrance into the intended target site, and altered intended or cellular activities as a result of mutation are the three main mechanisms of resistance in *A. baumannii* (Lowe et al., 2018). The World Health Organization has designated Carbapenem-resistant *A. baumannii* as a priority-I pathogen, necessitating the development of new antimicrobial agents (Ingti et al., 2020; Khurshid et al., 2020). Because of this, there is a desperate search for new antimicrobial drugs that can inhibit virulent targets and research the function of possible medicines in the treatment of illnesses brought on by MDR *A. baumannii* (Shahzad et al., 2020)*.*

As a result, the lack of antimicrobial therapeutics that are effective in treating MDR organisms is leading to the urgent need for new options that may be more effective in treating MDR phenotypes (Chen et al., 2020). Mur ligases seem to have become the focus of antibacterial therapy research in recent years (Schneider and Sahl, 2010; Bugg et al., 2011). Several classes of MurF inhibitors have been proposed (Sobral et al., 2003; Baum et al., 2006; Comess et al., 2006; Baum et al., 2007). Based on structure-based virtual screening, a novel inhibitor against *Streptococcus pneumoniae* MurF was revealed (Turk et al., 2009). A series of computational biology techniques ranging from molecular modelling and docking to binding free energy calculation and molecular dynamics simulations which determine the binding potential of various antibacterial towards AdeB, AcrB, and NorM efflux proteins were reported (Verma et al., 2018). Studies have suggested that several natural inhibitors can potentially bind and inhibit various key enzymes or major receptors which resulted in the inhibition of bacterial growth making them viable and attractive targets for screening of potent antibacterial drugs (Santajit and Indrawattana, 2016). Studies reported that alkaloids, flavones, tannins, and phenolic compounds are known to be active against various targets of *Acinetobacter spp*. (Liu et al., 2018). Recently few computational studies identified Mur members (MurA and MurG) as potential drug targets for MDR *A. baumannii* and *M. tuberculosis*, respectively (Saxena et al., 2018; Skariyachan et al., 2019).

Because additional NPs with inhibitory activity against MurF of MDR *A. baumannii* are yet to be explored to produce a new potential drug against *A. baumannii*. Although microbial natural products from soil origin have been widely explored, the metabolites of those occupying some special ecological niches have not been fully investigated. The ability of insects to live in unique niche habitats is often facilitated by the association with their microbial symbionts (Zhang et al., 2008; Brownlie and Johnson, 2009; Zhang et al., 2013). Termites are the major eusocial insect and play an important role in carbon and nitrogen cycles on earth (Ellwood and Foster, 2004). A Fungus Growing termite is a special group, belonging to *Macrotermitinae*, which form a close relationship with *Termitomyces* in the nest, besides having symbiosis with diverse gut microbes (Aanen et al., 2007; Rosengaus et al., 2010). Fungus-growing termite usually has higher efficiency of lignocellulose decomposition (Poulsen, 2015). Fungal-growing termite gut harbours a diverse and varied microflora that is indispensable for multifunctional niches, additionally, the tripartite symbiosis in fungus-growing termites may foster some special microbes with unique metabolic mechanisms, which may provide important resources for discovering novel antimicrobial drugs (Otani et al., 2019). Fungus growing termites could be termed as biopharma in terms of a major source of antimicrobials, as it has a huge bio potential because of the enormous amount of microflora inside its gut yielding important chemical scaffolds urgently needed for novel natural products and future drug development programs. Natural compounds derived from the fungus-growing termite are of particular interest to medicinal chemists owing to their outstanding chemistry and varied biological characteristics. *Actinobacteria* are good potential defensive symbionts in fungus-growing termites because they are well-known antibiotic generators and occur as defensive microbes in other insect–fungus symbioses (Scott et al., 2008; Kroiss et al., 2010; Seipke et al., 2011). Termite nests had huge Actinobacteria diversity (Manjula et al., 2016) and housed more than 20% of Actinobacteria that could inhibit the growth of at least one tested organism (Sujada et al., 2014). It was also reported that some Actinobacteria from the faecal nest of active termites had antimicrobial activity (Chouvenc et al., 2013).

Considering the importance of MurF, we purposefully constructed a small library of new ecological niche, fungus-growing termite-associated NPs (n = 376) to identify lead molecules that may act as inhibitors for the MurF (PBD ID: 4ZIY) protein of MDR *A. baumannii.* Subsequently, screened Fungus-growing termite-derived NPs (n = 376) were subjected to screening for their physicochemical characteristics, pharmacokinetics, and drug-likeness. The leading 74 natural products (with no Lipinski’s rule of five violation) were docked against MurF protein, and the lead (based on top Gold score) four compounds Maduralactomycin A, Natalenamide C, Termstrin B (Anthraquinone derivative) and Fridamycin A were evaluated using different post-molecular dynamics analyses as a part of *in silico* modelling. Maduralactomycin A has the most favourable binding energies which make it a more important compound to warrant further investigation. Consequently, our results showed that these top drug candidates can serve as potential inhibitors of MurF protein based on their behaviour that is explored as a function of time.

## Materials and methods

### Crystal structure retrieval

The crystal structure of MurF (PDB ID: 4ZIY) was retrieved from the Protein Data Bank (PDB) and used as the template for the virtual screening of fungal-associated termite-derived natural products (NPs). The structures were downloaded in PDB format and viewed using the PyMOL software (DeLano, 2002). The structures were then prepared for docking by removing water molecules, ions, and other ligands, and then minimized the structure using the Schrodinger software with the OPLS4 force field (Lu et al., 2021) and the steepest descent algorithm until the gradient threshold of 0.01 kcal/mol/Å was reached.

### Library design and optimization

A library of 376 NPs was downloaded from the PubChem database (Kim et al., 2021) and screened for drug-like properties using Swiss ADME (Daina et al., 2017). The 74 leading NPs were then docked against the MurF protein using the CCDC GOLD suite (Jones et al., 1997). The structures were optimized using the Avogadro tool (Hanwell et al., 2012) and Chem3D (Chem3D, 1991). The Avogadro tool was used to perform energy minimization of the structures using the MMFF94x force field (Halgren, 1996) and the steepest descent algorithm until the gradient threshold of 0.001 kcal/mol/Å was reached. Chem3D was used to perform conformational searches on the structures using the Molecular Mechanics Poisson-Boltzmann Surface Area (MM-PBSA) method (Cousins, 2005) with a total of 1000 conformations generated for each structure.

### Computational pharmacokinetics

A substantial bottleneck remains in the drug development approaches, especially in the later stages of lead NPs discovery. ADME profile analysis and explicit toxicity features of a drug-like candidate can compensate for the technological gaps and difficulties (Lee and Goo, 2004). Using Lipinski rule-based ADME criteria, compounds are evaluated as potential to be used as an effective therapeutic option (Lipinski et al., 1997). The SWISS-ADME tool was used to determine a set of ADME-related attributes (Rashid et al., 2021; Mohammed Ali et al., 2022) for each of the selected NPs. The ADME properties of the 74 NPs were analyzed using the SWISS-ADME tool (Daina et al., 2017) to evaluate their drug-like suitability and toxicity risk. Lipinski’s rule of five (Lipinski et al., 1997) was used to predict the partition coefficient (Log P), H-bond donors and acceptors, topological polar surface area (TPSA), number of rotatable bonds, molecular weight, and number of atoms. The predictions were made using the default parameters of the SWISS-ADME tool with a cut-off threshold of 3 for Log P and TPSA and a cut-off threshold of 5 for H-bond donors and acceptors. While looking for the drug-likeness, toxicity risk predictor is of utmost importance. Toxicology risk alerts are a sign that the depicted structure might be detrimental in relation to the given risk category. Risk alarms, however, are not intended to be a perfectly accurate toxicity prediction (Rashid et al., 2021).

### Molecular docking

The MurF protein was configured for docking using the Hermes visualizer in the CCDC GOLD suite (Jones et al., 1997). The binding site of the crystal ligand was used to identify the active site of the protein. The GOLD scoring function (goldscorep450_csd) was used to rank the docked NPs based on their binding affinity. The four best-docked NPs were selected for further analysis. The docking calculations were performed using the default parameters of the CCDC GOLD suite with a grid box size of 30x30x30 Å and a grid spacing of 0.375 Å.

### Explicit solvent molecular dynamics simulation

The Desmond program of Schrodinger software (version 2021-2) (Bowers, 2006) was used for the explicit solvent molecular dynamics simulation of the selected NPs. The OPLS4 force field (Jorgensen et al., 1996) was used to model protein-ligand interactions. A simulated triclinic periodic boundary box with an extension of 10 Å in each direction was made to help solve the structures of the *A. baumannii* MurF target protein. Explicit solvation models (Monte-Carlo equilibrated SPC, the transferable intermolecular potential 3 points) were used for each system. (Jorgensen et al., 1983). Lennard Jones (LJ) interactions (with cut-off value = 10) (Shaik et al., 2010) and the SHAKE algorithm (Kräutler et al., 2001) were applied to standardize the mobility of all Bonds (covalent and hydrogen bonds). The system was solvated with additional counter ions (0.15 M of Na + Cl) to neutralize the system. The protein-ligand complex was energy minimized using the steepest descent algorithm until the gradient threshold of 25 kcal/mol/Å was reached. The complex was subjected to molecular dynamics simulation in NPT ensemble class at 300 K and 1 bar pressure using default parameters of the Desmond program. The simulation was run for a total of 200 nanoseconds, with a time step of 2 fs, and data were collected every 20 ps.

### Prime MMGBSA calculation

The Schrodinger software (version 2021-2) was used to perform Prime MMGBSA calculations on the selected NPs. The OPLS4 force field (Jorgensen et al., 1996) was used to model protein-ligand interactions. The complexes were solvated in an explicit solvent and energy was minimized. The binding free energy of the complexes was calculated using the Prime MMGBSA method (Jacobson et al., 2002) and used to rank the NPs based on their binding affinity to the MurF protein. The calculations were performed with the default parameters of the Schrodinger software, including the use of the GB/SA implicit solvent model, a dielectric constant of 1.0, and a scaling factor of 0.8 for van der Waals interactions. A total of 100 frames were obtained from all the trajectories at a uniform time period. The final energies were calculated based on the average energies from these 100 frames. The binding free energy of a ligand (L) to a protein (P) to form the complex (PL) is obtained as the difference.

$$\Delta G_{\left(binding\right)}=\Delta G_{\left(complex\right)}-\Delta G_{\left(Protein\right)}-\Delta G_{\left(Ligand\right)}$$

Where ∆G _*(complex)*_, ∆G _*(Protein)*_, and ∆G _*(Ligand)*_ stand for the free energies of a complex, a protein, and a ligand, respectively while ∆G _*(binding)*_ is the binding free energy.

## Results

### Structure preparation and screening of fungus-growing termite derived NPs

**FIGURE 1:**
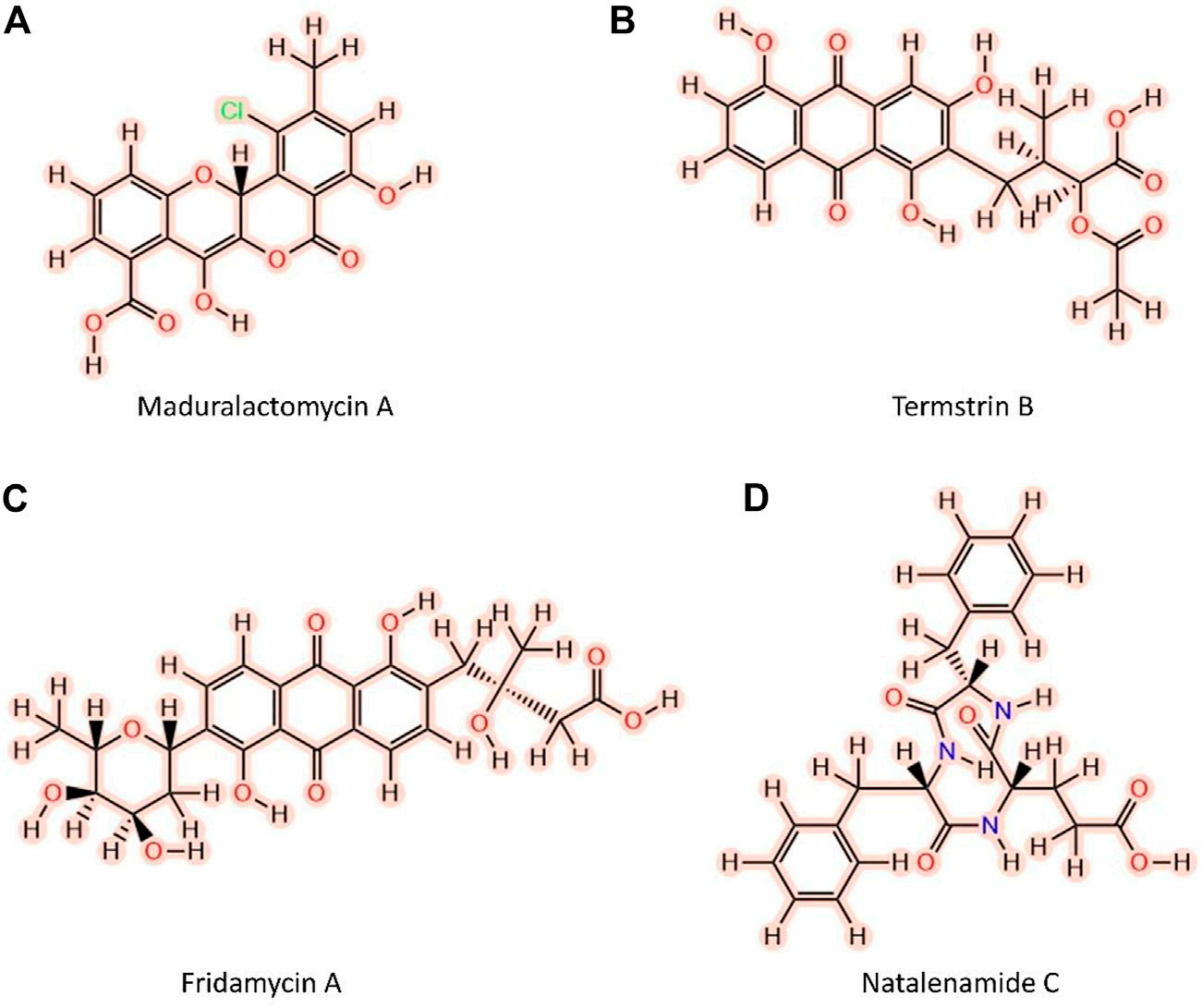
Two-dimensional structures of the top four Fungus-growing termite-derived NPs against MurF protein of *A. baumannii* shortlisted after molecular docking and computational Pharmacokinetics. **(A)** Maduralactomycin A, **(B)** Termstrin, **(C)** Fridamycin A, **(D)** Natalenamide C.

The three-dimensional structure of the MurF protein of A. baumannii was obtained from a protein databank using the PDB ID 4ZIY. The structure was cleaned off irrelevant protein chains and crystallization waters. The cleared structure was energy minimized for 3000 steps using the steepest descent and conjugate gradient algorithms (1500 each). The active site information was extracted from the bound crystallographic MurF structure. A small library of 2D structures of 376 Fungus-growing termite-derived NPs was developed, and the structures were converted to 3D using the chem3d tool. The 3D structures were further optimized using the Avogadro tool to find the minimum energy structure of each NP. The MMFF94 force field (Halgren, 1996) was used for energy minimization and the number of iterations was set to 500 and the gradient convergence threshold was tuned to 0.05 kcal/mol/Å. Van der Waals cutoff distance was set to 8 Å and the electrostatics cutoff distance was set to 10 Å to get the more reliable geometry of the compounds. For screening against the MurF protein of the *A. baumannii,* CCDC Gold Suite was utilized. Since the active site contains a metal ion, we utilized GoldScore as a scoring function which takes into account chemscore, metal-ligand interactions and along with the entropic cost of the ligand desolvation. The genetic search algorithm (GA) with maximum GA operations of 100, the maximum number of solutions set to 10 and 100 conformations per ligand with Van der Waals, Electrostatic, Hydrogen bond, and metal-binding scaling factor set to 1.0. The ligand flexibility was set to rigid and receptor flexibility was set to the rigid backbone and flexible sidechains. The Fungus-growing termite derived NPs library was screened, and a gold score was obtained for all the compounds. After searching for the drug-likeness and Goldscore collectively, four compounds with an established history of extraction from Fungus-growing termites were selected as lead candidates for further study (Figure 1; Table 1).

**TABLE 1:** The table enumerates the shortlisted NPs derived from Fungus-growing termites (novel ecological niche).

S.No	Ligand	Compound class	Termite source	Bacterial source	Ref.
1	Termstrin B	Anthraquinone derivative	*O. formosanus*	*Streptomyces tanashiensis*	Zhang et al. (2020)
2	Fridamycin A	PKS	*Macrotermes natalensis*	*Actinomadura* sp. *RB99*	Yoon et al. (2019)
3	Maduralactomycin A	PKS	*Macrotermes natalensis*	*Actinomadura* sp. *RB29*	Guo et al. (2020)
4	Natalenamide C	NRPS	*Macrotermes natalensis*	*Actinomadura* sp. *RB99*	Lee et al. (2018a)

Remarks: PKS; polyketide synthases, NRPS; Non-Ribosomal Peptide Synthetase.

### Lipinski validation of the lead NPs

Lipinski Rules: Lipinski’s rule of five is a set of guidelines used to predict the oral bioavailability of a drug candidate. The four rules state that a compound should have no more than 5 hydrogen bond donors (HBD), no more than 10 hydrogen bond acceptors (HBA), a molecular weight of less than 500, and an octanol-water partition coefficient (logP) of less than 5. Termstrin B has 2 HBD, 9 HBA, a molecular weight of 412.35, and a logP of 2.44, which means it has 0 violations of Lipinski’s rules. Fridamycin A has 5 HBD, 10 HBA, a molecular weight of 485.46, and a logP of 1.6, which means it has 0 violations of Lipinski’s rules. Maduralactomycin A has 1 HBD, 7 HBA, a molecular weight of 352.29, and a logP of 2.21, which means it has 0 violations of Lipinski’s rules. Natalenamide C has 3 HBD, 5 HBA, a molecular weight of 422.45, and a logP of 1.98, which means it has 0 violations of Lipinski’s rules (Table 2).

**TABLE 2:** The table shows the various drug-like properties of the top four compounds including Lipinski’s rule of five, solubility, GI absorption, blood-brain permeation bioavailability score, brink alerts and synthetic accessibility, etc. For lead compounds.

ADME and toxicity profiling of shortlisted fungus-growing termite derived NPs				
	Maduralactomycin A	Fridamycin A	Termstrin B	Natalenamide C
PubMed CID	156582985	75614470	156580654	146684096
Physiochemical Property				
Formula	C18H11ClO7	C25H26O10	C21H18O9	C23H25N3O5
Molecular Weight	374.73	486.47	414.36	423.46
Num. H-bond donors	3	6	4	4
Num. H-bond acceptors	7	10	9	5
Num. Rotatable bonds	1	5	6	7
TPSA	113.29	181.82	158.43	124.6
Molar Refractivity	90.29	121.26	102.68	124.37
ADME Daina et al. (2017)				
BBB permeate	No	No	No	No
GI absorption	High	Low	Low	High
Pgp substrate	No	Yes	Yes	Yes
Brenk #alerts	0	0	0	0
Silicos-IT class	Moderately soluble	Soluble	Soluble	Poorly Soluble
Ali Class	Moderately soluble	Moderately soluble	Poorly soluble	Soluble
CYP1A2 inhibitor	Yes	No	No	No
CYP2C19 inhibitor	No	No	No	No
CYP2C9 inhibitor	Yes	No	Yes	No
CYP2D6 inhibitor	Yes	No	No	No
CYP3A4 inhibitor	Yes	No	No	No
log Kp (cm/s)	−6.29	−8.13	−6.36	−8.3
Lipinski violations	0	0	0	0
Ghose violations	0	1	0	0
Veber violations	0	1	1	0
Egan violations	0	1	1	0
Muegge violations	0	1	1	0
Bioavailability Score	0.56	0.11	0.11	0.56
Synthetic Accessibility	4.18	5.15	3.99	4.02
Molinspiration Bioactivity Score Molinspiration Log. (2017)				
GPCR ligand	−0.38	0.01	0.14	0.33
Ion channel modulator	−0.52	−0.01	0.08	0.13
Kinase inhibitor	−0.47	−0.06	−0.01	−0.01
Nuclear receptor ligand	0.06	−0.11	0.55	0.16
Protease inhibitor	−0.53	−0.2	0.26	0.47
ProtoxII Prediction of toxicity Banerjee et al. (2018)				
Predicted Toxicity Class	Yellow	Yellow	Yellow	Yellow
Hepatotoxicity	Inactive 61%	Inactive 72%	Inactive 67%	Inactive 68%
Carcinogenicity	Medium Risk	Inactive 64%	Inactive 75%	Inactive 74%
Mutagenicity	Inactive 56%	Inactive 60%	Inactive 55%	Inactive 82%
Cytotoxicity	Inactive 68%	Inactive 79%	Inactive 81%	Inactive 78%
Estrogen Receptor Alpha (ER	Inactive 59%	Inactive 76%	Inactive 53%	Inactive 87%
Phosphoprotein (Tumor Suppressor) p53	Medium Risk	Medium Risk	Inactive 71%	Inactive 87%
Gold Score against MurF PBD ID: 4ZIY	68.69	76.6	65.25	68.13

Remarks: Molinspiration Bioactivity Score > 0—active; −5.0–0.0—moderately active; < −5.0—inactive.

### Drug likeness of the top compounds

The compound Termstrin B has a TPSA (topological polar surface area) of 164.09. According to the Ali Log S, the solubility of this compound is 1.02E-04 mg/mL and 2.46E-07 mol/L, which is classified as “poorly soluble”. According to the Silicos-IT LogSw, the solubility of this compound is 7.05E-02 mg/mL and 1.71E-04 mol/L, which is classified as “soluble”. The compound has low GI absorption, log Kp of −6.34, no Lipinski violations, a bioavailability score of 0.11, no Brenk alerts, and synthetic accessibility of 3.92. The compound Fridamycin A has a TPSA of 184.65. According to the Ali Log S, the solubility of this compound is 3.95E-03 mg/mL and 8.14E-06 mol/L, which is classified as “moderately soluble”. According to the Silicos-IT LogSw, the solubility of this compound is 1.85E-01 mg/mL and 3.82E-04 mol/L, which is classified as “soluble”. The compound has low GI absorption, log Kp of −8.13, no Lipinski violations, a bioavailability score of 0.11, no Brenk alerts, and synthetic accessibility of 5.11. The compound Maduralactomycin A has a TPSA of 118.95. According to the Ali Log S, the solubility of this compound is 2.60E-03 mg/mL and 7.39E-06 mol/L, which is classified as “moderately soluble”. According to the Silicos-IT LogSw, the solubility of this compound is 2.92E-02 mg/mL and 8.28E-05 mol/L, which is classified as “moderately soluble”. The compound has high GI absorption, log Kp of −6.34, no Lipinski violations, a bioavailability score of 0.56, no Brenk alerts, and synthetic accessibility of 4.18. This last compound Natalenamide C has a TPSA of 127.43. According to the Ali Log S, the solubility of this compound is 3.53E-01 mg/mL and 8.35E-04 mol/L, which is classified as “soluble”. According to the Silicos-IT LogSw, the solubility of this compound is 1.05E-04 mg/mL and 2.48E-07 mol/L, which is classified as “poorly soluble”. The compound has low GI absorption, log Kp of −8.29, no Lipinski violations, a bioavailability score of 0.56, no Brenk alerts, and synthetic accessibility of 3.98. It is important to note that these computationally predicted properties are not always accurate and experimental validation is needed before reaching a conclusion.

### Molecular docking analysis

The shortlisted compounds exhibited favourable gold scores against the MurF protein of *A. baumannii*. Compound Termstrin B posted the highest gold score of 76.99 followed by Fridamycin A at 76.6. The Maduralactomycin A and Natalenamide C posed a gold score of 68.69 and 68.13 (Table 3).

**TABLE 3:** The table lists the molecular docking scores, hydrogen interactions of the candidate drug and protein as well as the hydrogen interactions of the Mg ion within the active site.

S.No	Ligand	Gold Score (MurF;4ZIY)	Hydrogen Interactions	MG interactions
1	Termstrin B	76.99	Ser117, Thr120, Thr121, Asn144 (2), Asn284, Asn287, Tyr334, Asn335	Thr120, Glu166, Termstrin B (3)
2	Fridamycin A	76.6	Ser117, Thr120, Asn142, Asn144, His283, Arg318, Asp332 (2), Asn335, Lys429	Thr120, Glu166 (2), Fridamycin A (2)
3	Maduralactomycin A	68.69	Lys119, Thr120 (3), Thr121 (4), Asn144, Tyr334, Asn335	Thr120, Glu166, Maduralactomycin A (2)
4	Natalenamide C	68.13	Thr120, Arg318, Asn335, Ser340	Thr120, Glu166 (2), Natalenamide C (2)

**FIGURE 2:**
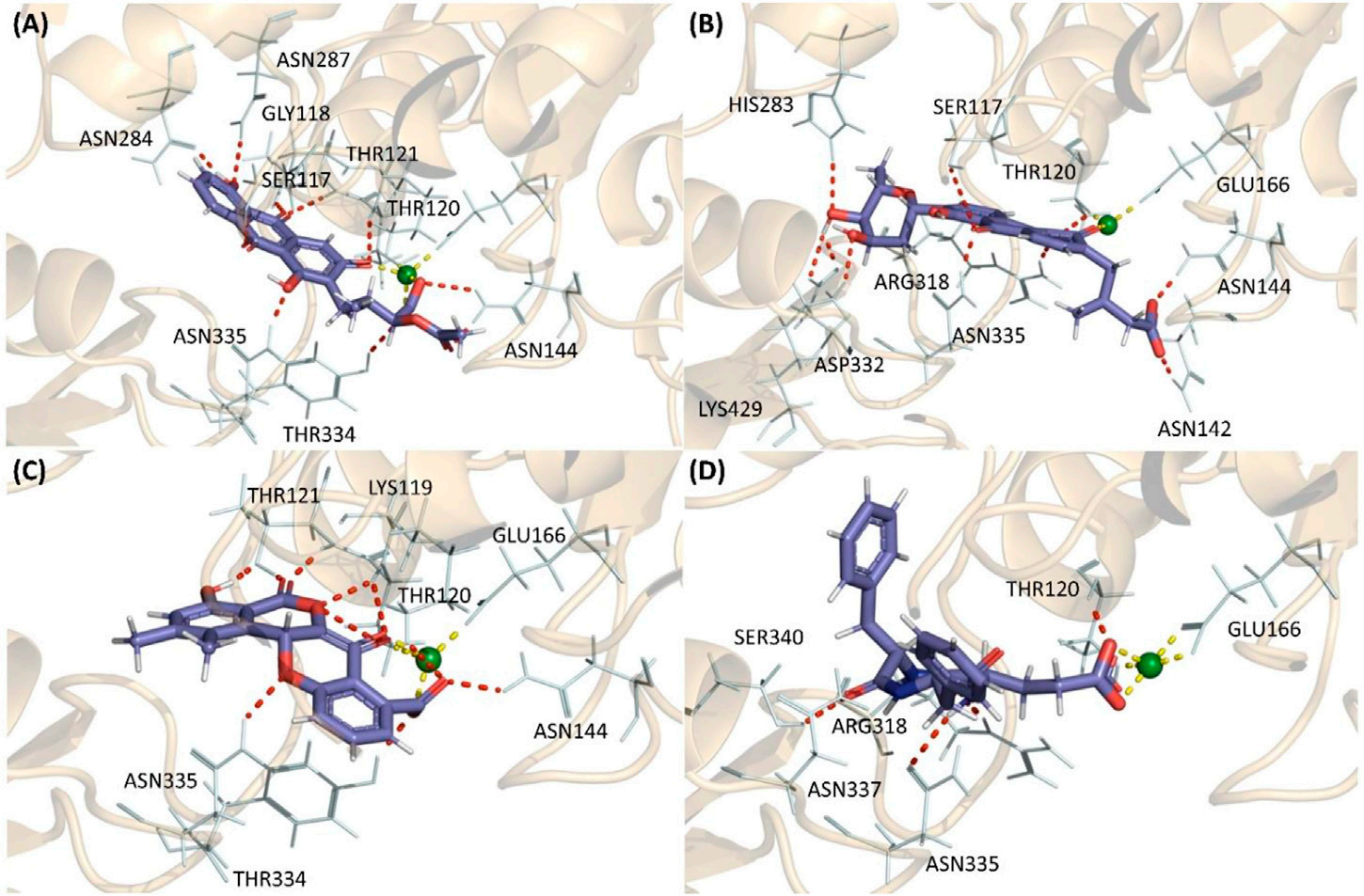
The highest scoring molecular docking poses of **(A)** Termstrin B **(B)** Fridamycin A **(C)** Maduralactomycin A, and **(D)** Natalenamide C bound to the active site of the MurF protein of A. baumannii. The hydrogen bonds between compounds and the protein residues/Mg ion are depicted as a red dotted line while hydrogen interactions among Mg ion and the protein residues are depicted via a yellow dotted line.

These higher gold scores were supported by the hydrogen bond interactions and hydrophobic interactions with the complexes mainly stabilized by the magnesium (Mg) ion. All the compounds established hydrogen interaction with Thr120 and Asn335 while the Mg ion established hydrogen bonds with Thr120, Glu166, and at least two interactions with the ligand in the binding pocket for all four complexes. This shows that all four compounds docked into the same active site in all four cases. Additionally, Termstrin B posed hydrogen bonds with Ser117, Thr121, Asn144 (2), Asn284, Asn287, and Tyr334 and Fridamycin A was stabilized by hydrogen interactions with Ser117, Asn142, Asn144, His283, Arg318, Asp332 (2), Lys429. While Maduralactomycin A exhibited hydrogen interactions with Lys119, Thr121 (4), Asn144, and Tyr334 with three hydrogen bonds with Thr120. The compound Natalenamide C posed hydrogen interactions with Arg318 and Ser340 (Figure 2).

### Maduralactomycin A is the most stable NP against the MurF of *A. baumannii*

**FIGURE 3:**
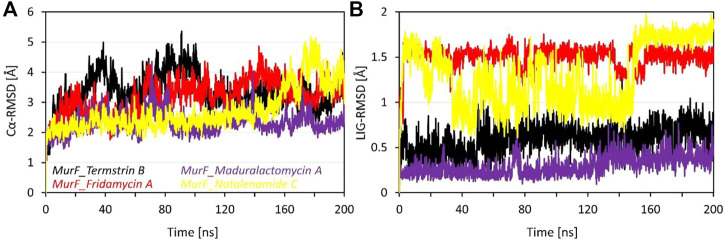
Root means square deviation analysis of the MurF protein of *A. baumannii* after 200 ns of molecular dynamics simulations. Where **(A)** represents the line RMSD graph for Cα atoms of MurF protein while **(B)** represents the RMSD values of each compound with respect to its initial position within the active site. The line graph is enriched with colour-coded legends for each complex.

The RMSD data for four compounds, i.e., Termstrin B, Fridamycin A, Maduralactomycin A, and Natalenamide C, bound to the MurF protein of *A. baumannii* was obtained after 200 ns molecular dynamics simulations. The analysis of the data shows that Maduralactomycin A has the lowest average RMSD value of 2.31 Å, while Termstrin B has the highest average RMSD value of 3.35 Å (). This suggests that Maduralactomycin A may have a more stable interaction with the MurF protein compared to the other compounds. The maximum RMSD values for each compound also indicate that the RMSD values for each compound increased throughout the simulation, and the highest RMSD values were observed at different time points, Termstrin B has the highest RMSD at 91ns and Natalenamide C has the highest RMSD at 177th ns. The standard deviation values indicate that the RMSD values for each compound are relatively consistent, with Maduralactomycin A having the lowest standard deviation of 0.35, while Termstrin B and Natalenamide C have the highest standard deviation of 0.58 and 0.70 respectively. It is worth noting that the difference between the average RMSD of Maduralactomycin A and Termstrin B is quite high, which suggests that Maduralactomycin A has a more stable interaction with the MurF protein than the other compounds (Figure 3). Additionally, the standard deviation of Maduralactomycin A is significantly lower than the other compounds, indicating that the structural stability of Maduralactomycin A is more consistent over the simulation. Furthermore, when analyzing the RMSD values concerning the initial position of the compounds in the active site of the MurF protein, Maduralactomycin A again stands out with the lowest average RMSD value of 0.29 Å, the lowest maximum RMSD value of 0.76 Å, and the lowest standard deviation of 0.10, further reinforcing its structural stability in the active site of the MurF protein. On the other hand, Fridamycin A has the highest average RMSD value of 1.50 Å, the highest maximum RMSD value of 1.75 Å, and the second-highest standard deviation of 0.11, indicating that it may have a less stable interaction with the active site of the MurF protein. Overall, the results of this RMSD analysis indicate that Maduralactomycin A is the most stable compound against the MurF of *A. baumannii*.

### Flexibility and β-factor analysis

**FIGURE 4:**
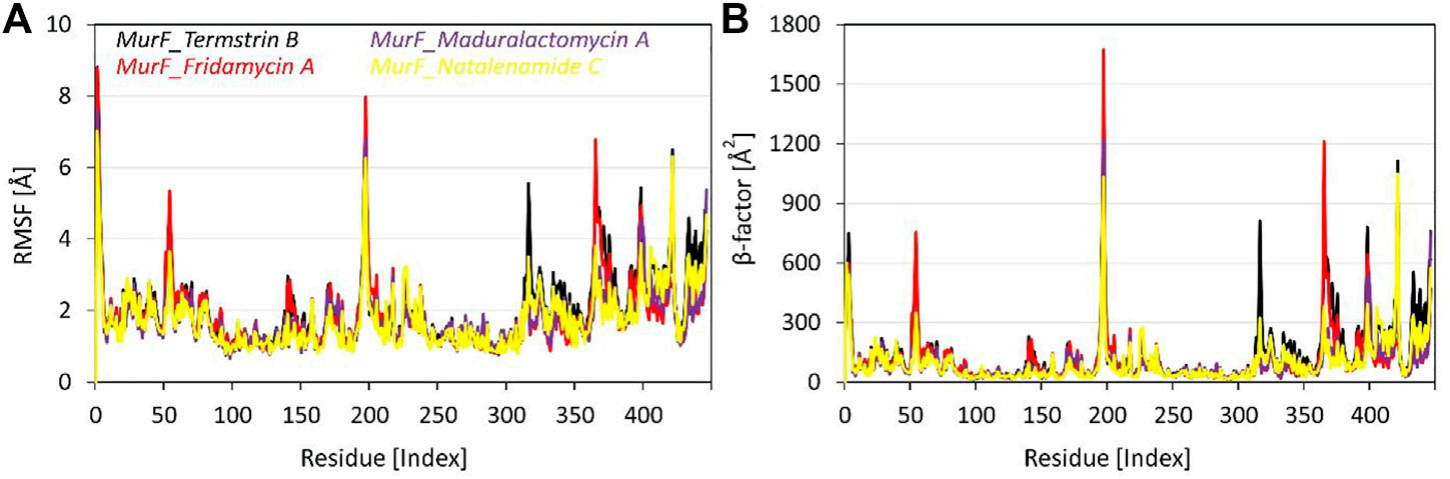
Root means square fluctuation and β-factor analysis of the MurF protein of *A. baumannii* after 200 ns of molecular dynamics simulations. Where **(A)** represents the line RMSF graph for residues of MurF protein while **(B)** represents the βfactor values of each residue while bound to the candidate compounds within the active site. The line graph is also enriched with colour-coded legends for each complex.

The RMSF analysis of the four compounds MurF protein (when bound to Termstrin B, Fridamycin A, Maduralactomycin A, and Natalenamide C) of *A. baumannii* was obtained after 200 ns molecular dynamics simulations. The analysis of the data shows that the residues Glu6, Pro7, and Trp8 are the most rigid residues across all four compounds, with RMSF values ranging between 1.39 Å to 1.75 Å. This suggests that these residues play a critical role in maintaining the structural stability of the complex. Moreover, other regions with high degrees of flexibility include residues 310-325, 358-370, and 418-426, with specific residues such as Gly313, Leu314, Gly316, and Gln317 in the first region, Asn359, Ser361, Leu364, and Ser365 in the second region, and Pro419, Leu420, Gln421, and Ser423 in the third region showing particularly high RMSF values. In the middle, the four residues with the highest RMSF values are Glu194, Asp195, Ser196, and Arg197. These residues may have a significant impact on the binding stability of the ligands. Additionally, the analysis also reveals three other regions of high flexibility: residues 310-320, 358-365, and 418 to 421. In the region of 310-325, the top four most flexible residues are Tyr314, Lys317, Arg318, and Asp319. In the region of 358-370, the top four most flexible residues are Tyr359, Val360, Glu362, and His364. Lastly, in the region of 418–426, the top four most flexible residues are Glu418, Asp419, Ser420, and Arg421 (Figure 4). These regions may also play a significant role in the binding and interaction of the compounds with the MurF protein. Overall, the RMSF analysis highlights the importance of the active site and surrounding regions in the binding and interaction of the compounds with the MurF protein. The Maduralactomycin A compound has the best overall performance in terms of RMSF values.

When comparing the β-factor data to the RMSF data, it is clear that the regions of high flexibility identified by RMSF analysis are also reflected in the β-factor data. For example, the RMSF analysis identified the active site residues Glu194, Asp195, Ser196, and Arg197 as having the highest RMSF values, and the β-factor data also shows that these residues have high β-factor values. Similarly, the RMSF analysis identified residues 310-320, 358-365, and 418-422 as regions of high flexibility, and the β-factor data also shows that these regions have high β -factor values. Additionally, the RMSF analysis identified the residues Glu6, Pro7, and Trp8 as the most rigid residues, and the β-factor data also shows that these residues have low β-factor values. Overall, the comparison of the RMSF and β-factor data supports the conclusion that the active site and surrounding regions play a significant role in the binding and interaction of the compounds with the MurF protein.

### Gyration and exposed surface area analysis

The radius of gyration (Rg) values provides an insight into the size and shape of the compounds in complex with the MurF protein from *A. baumannii*. The average Rg values for MurF bound Termstrin B, Fridamycin A, Maduralactomycin A and Natalenamide C were 25.31 Å, 25.47 Å, 25.04 Å and 25.13 Å, respectively which are relatively similar, indicating that the induced compactness by the size and shape and activity of these compounds are similar. The standard deviation (SD) of the Rg values is 0.31 Å, 0.27 Å, 0.26 Å, and 0.37 Å for Termstrin B, Fridamycin A, Maduralactomycin A and Natalenamide C, respectively, which are relatively small, indicating that there is not much variation in the Rg values within each complex. The maximum Rg value for Termstrin B was 26.24 Å, 26.38 Å for Fridamycin A, 26.07 Å for Maduralactomycin A and 26.18 Å for Natalenamide C while the minimum Rg values were 24.33 Å, 24.48 Å, 24.34 Å and 24.12 Å, respectively. In comparison, the Rg values for Maduralactomycin A are slightly lower than the other compounds which may suggest that Maduralactomycin A has induced a more compact protein structure complex as compared to the other compounds.

**FIGURE 5:**
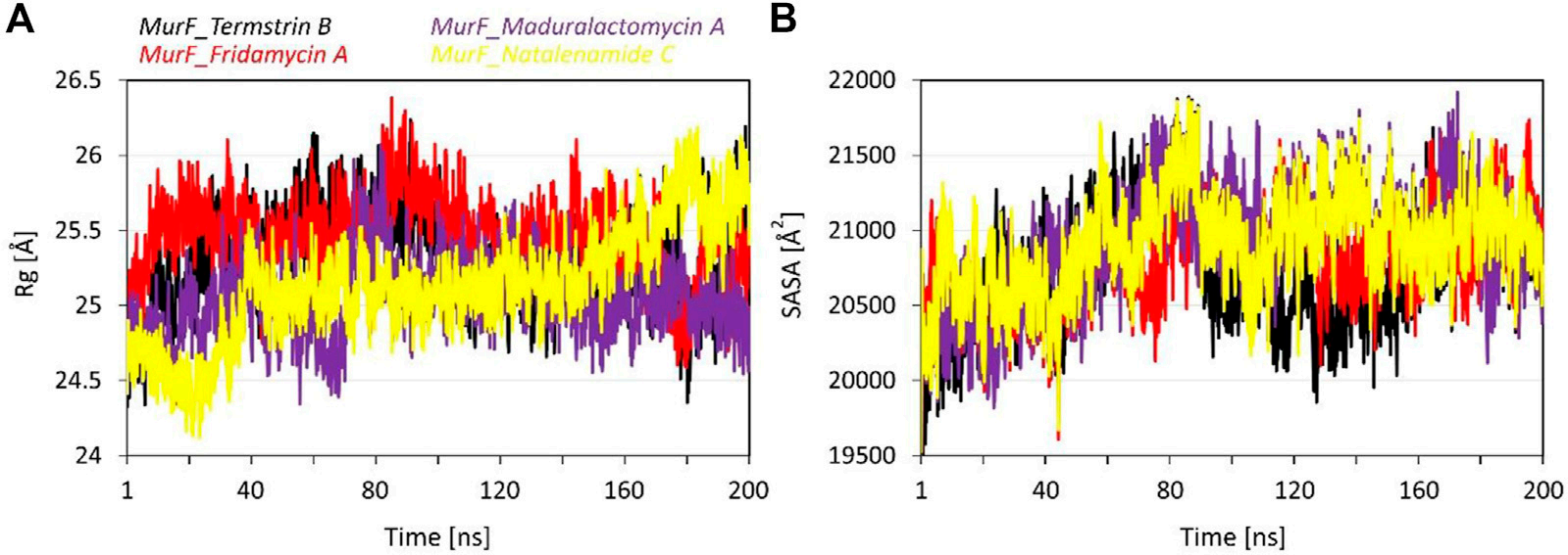
The line graph shows **(A)** the Radius of gyration (in Å), and **(B)** Solvent accessible surface area (in Å^2^) plots as a function of time for the simulation period of 200 ns per system. The line graph has been enriched with colour-coded legends for each graph.

The results of the SASA analysis provide information on the exposed surface area of the compounds in complex with the protein. The Average SASA induced by Termstrin B was 20747.86 Å^2^, 20821.24 Å^2^ for Fridamycin A, 20907.37 Å^2^ for Maduralactomycin A, and 20902.57 Å^2^ for Natalenamide C. These values indicate that the compounds have a relatively similar exposed surface area within the binding pocket of the protein. However, the variability of the exposed surface area is higher for Termstrin B (376.46) and Maduralactomycin A (378.73) as compared to Fridamycin A (310.44) and Natalenamide C (327.43). This suggests that the exposed surface area of these compounds may be more variable within the binding pocket of the protein as compared to the other compounds. The maximum SASA values are 21888.80 Å^2^ for Termstrin B, 21734.86 Å^2^ for Fridamycin A, 21918.81 Å^2^ for Maduralactomycin A, and 21873.80 Å^2^ for Natalenamide C while the minimum SASA values are 19432.83 Å^2^, 19528.59 Å^2^, 19499.54, and 19525.54 Å^2^, respectively, which indicate that these compounds have a relatively similar range of exposed surface area (Figure 5). In conclusion, the SASA analysis suggests that all the compounds have similar surface exposure in complex with the MurF protein. However, it is worth noting that Maduralactomycin A has the highest maximum SASA value and Termstrin B has the lowest minimum SASA value, indicating that these compounds may have slightly different binding modes or conformations in the protein’s binding pocket.

### Intra-protein hydrogen bond and secondary structure analysis

The data provided for the hydrogen bonds analysis pertains to the number of inter-protein hydrogen bonds formed when the compounds are bound to protein. The average number of hydrogen bonds formed varies among the compounds, with Termstrin B induced the highest at 404.67 followed by Maduralactomycin A and Natalenamide C, both with an average of 393.67. Fridamycin A forms the least number of hydrogen bonds, with an average of 392.32. The SD of Fridamycin A having is the highest at 9.44 while Termstrin B has the lowest at 5.36. Termstrin B has the highest maximum number of hydrogen bonds (424) while Fridamycin A has the lowest (363). It is worth noting that the standard deviation of Fridamycin A is relatively high, indicating that there is greater variability in the number of inter-protein hydrogen bonds formed for this compound compared to the others. The number of inter-protein hydrogen bonds formed can be indicative of the strength and stability induced by interacting compounds within the protein complex.

**FIGURE 6:**
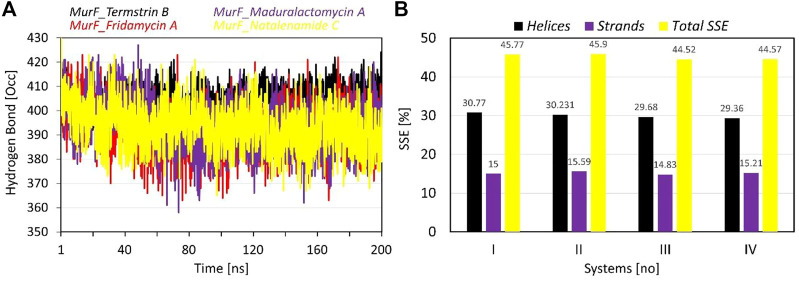
The plots show the results of **(A)** intra-protein hydrogen bond occupancy, and **(B)** Secondary structure elements (in percentages) analysis during the simulation time of 200 ns per system. The line graph has been enriched with colour-coded legends for each graph.

The analysis of secondary structural elements (SSEs) in the protein complex of interest provides insight into the local conformational changes that occur upon binding of the ligands. The SSEs are classified into two major categories: helices and strands. Figure 6 provides the percentage of helices and strands in each of the four complexes, as well as the total percentage of SSEs. It can be observed that there is a small difference between the four complexes in terms of the percentage of helices, which ranges from 29.36% to 30.77%. Similarly, the percentage of strands also varies slightly, with a range of 14.83%–15.59%. The total percentage of SSEs is also relatively consistent, with a range of 44.52%–45.9%. This suggests that the binding of the ligands does not lead to significant changes in the overall secondary structure of the protein. However, it should be noted that even small changes in SSEs may result in large changes in protein activity and binding. Overall, the SSE analysis provides a detailed picture of the structural changes that occur upon binding the ligands to the protein.

### MurF protein contacts with lead compounds

**FIGURE 7:**
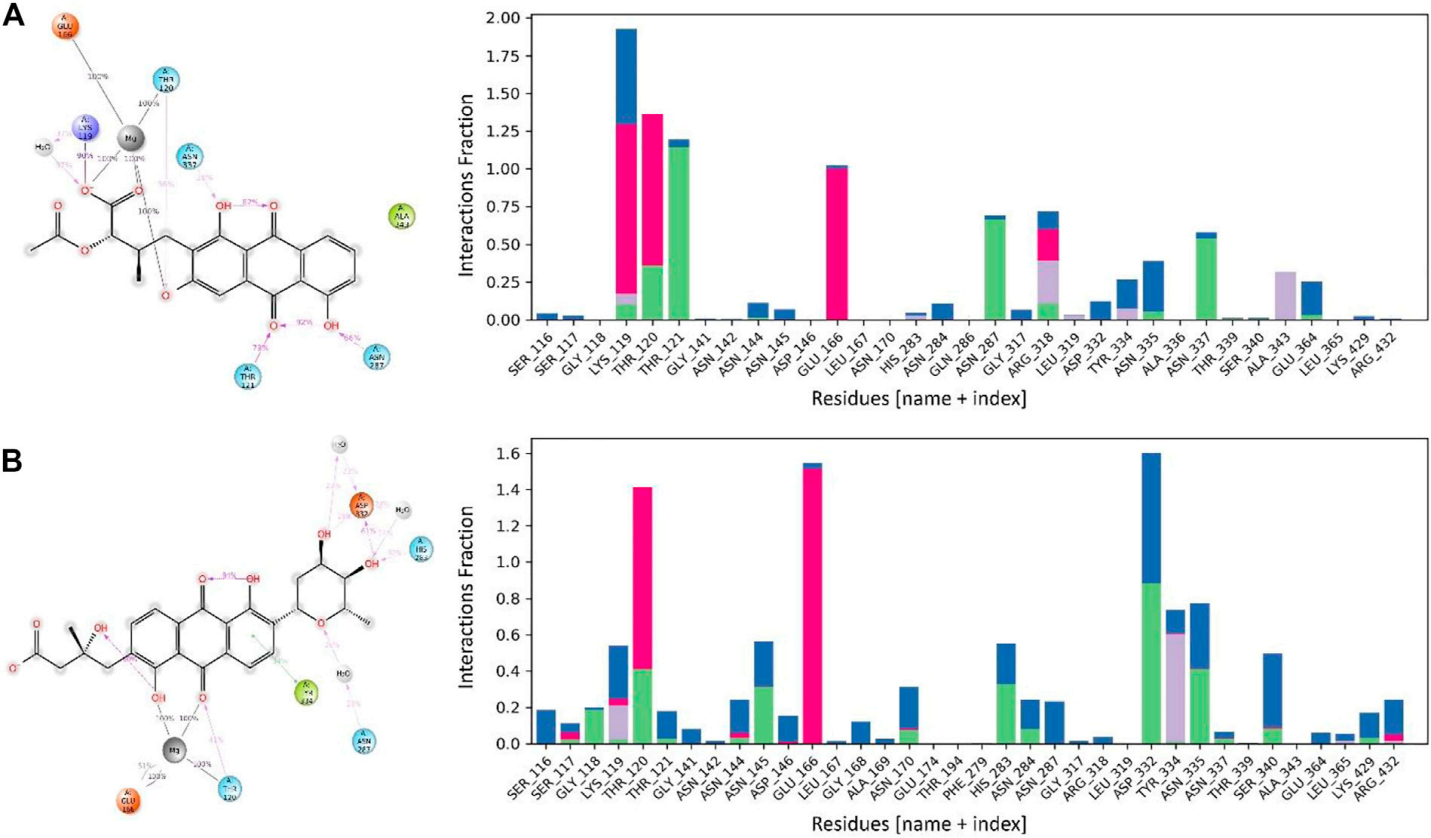
The interaction patterns and the fraction of MurF protein in complex with **(A)** Termstrin B and **(B)** Fridamycin A through the simulation period. The Histogram plot shows the fraction interaction of each residue, and the colour codes show the type of interaction, i.e., the green colour represents hydrogen bonds, the grey colour represents hydrophobic interactions, the purple colour represents ionic interactions, and the blue colour represents the water bridges.

The interactions between the MurF protein and the lead compounds were tracked for all four systems throughout the simulation time. It was observed that all the compounds were stabilized by the Mg ion within the active site of the MurF protein. Termstrin B retained three hydrogen bonds for 100% of the simulation time with the Mg ion while the hydrogen bonds between Thr120 and Glu166 and the Mg ion were also retained for the whole simulation time. Other interactions like the salt bridge between O^−^ of the Termstrin B and Lys119 were retained for 90% of the simulation time. Other notable retained interactions were between Termstrin B and Thr121 and Asn287 which were retained for 79% and 66% of the simulation time. The third major contributor for Termstrin B was a water bridge that was formed between the ligand and the Lys119 residue. On the other hand, for Fridamycin A, the Mg ion retained two interactions with the ligand for 100% of the simulation time while alternatively, the Mg ion retained two hydrogen bonds with Glu166 and Thr120 for 100% of the simulation period and 2nd bond with Glu166 for 51% of the time period. The second major contribution was made by Asp332 which retained two interactions for 61% and 26% respectively. Apart from that all other contributions were below 50% (Figure 7).

**FIGURE 8:**
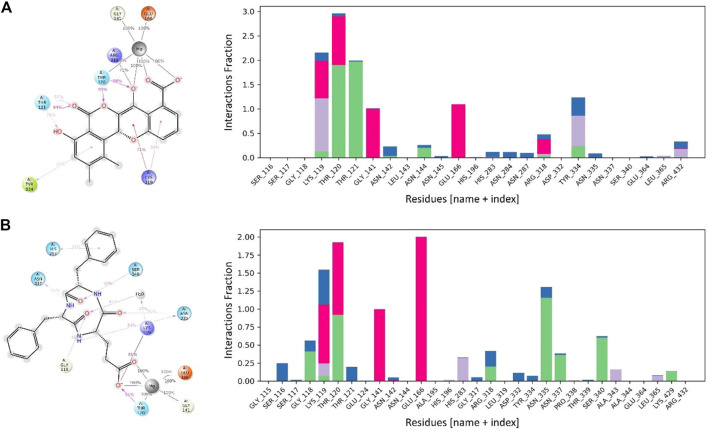
The interaction patterns and fractions of MurF protein in complex with **(A)** Maduralactomycin A and **(B)** Natalenamide C through the simulation period. The Histogram plot shows the fraction interaction of each residue, and the colour codes show the type of interaction, i.e., the green colour represents hydrogen bonds, the grey colour represents hydrophobic interactions, the purple colour represents ionic interactions, and the blue colour represents the water bridges.

Maduralactomycin A exhibited the three hydrogen bonds with Mg ion for 100% of the simulation period while Mg ion also retained two hydrogen bonds with Thr120, Gly141, and Glu166 for the whole simulation period. This shows the tight packing of Maduralactomycin A in the binding pocket of the MurF protein compared to the other compounds. Additionally, a major contribution was also made by Lys119, Thr120, Thr121, and Arg318 as Lys119 retained two π-cation interactions for 71% and 37%, Thr120 retained two hydrogen interactions for 95% and 86%, Thr121 retained three hydrogen interactions for 94%, 51%, and 38% while Arg318 retained a single salt bridge for 71% of the simulation period. An additional contribution was also made by Tyr334 as a π-π stacking interaction. Natalenamide C also exhibited strong binding patterns within the binding pocket by retaining three hydrogen bonds for the whole simulation time with Mg ion and Mg ion also retained two hydrogen bonds with Glu166 and one hydrogen bond each with Thr120 and Gly141 residues for the simulation period. An additional contribution was made by Lys119, Thr120, Asn335, and Ser340 as Lys119 made a salt bridge and water bridge for 81% and 47%, Thr120 retained a hydrogen bond for 91%, Asn335 exhibited two hydrogen bonds each for 54% and 40% while Ser340 exhibited a hydrogen bond with Natalenamide C for 59% of the simulation time (Figure 8). An additional small contribution was also made by a π-π stacking with His283 residue. These results show that Madulactomycin A and Natalenamide C exhibited the strongest binding against the MurF protein of *A. baumannii*.

### Binding energy (MMGBSA) analysis

**FIGURE 9:**
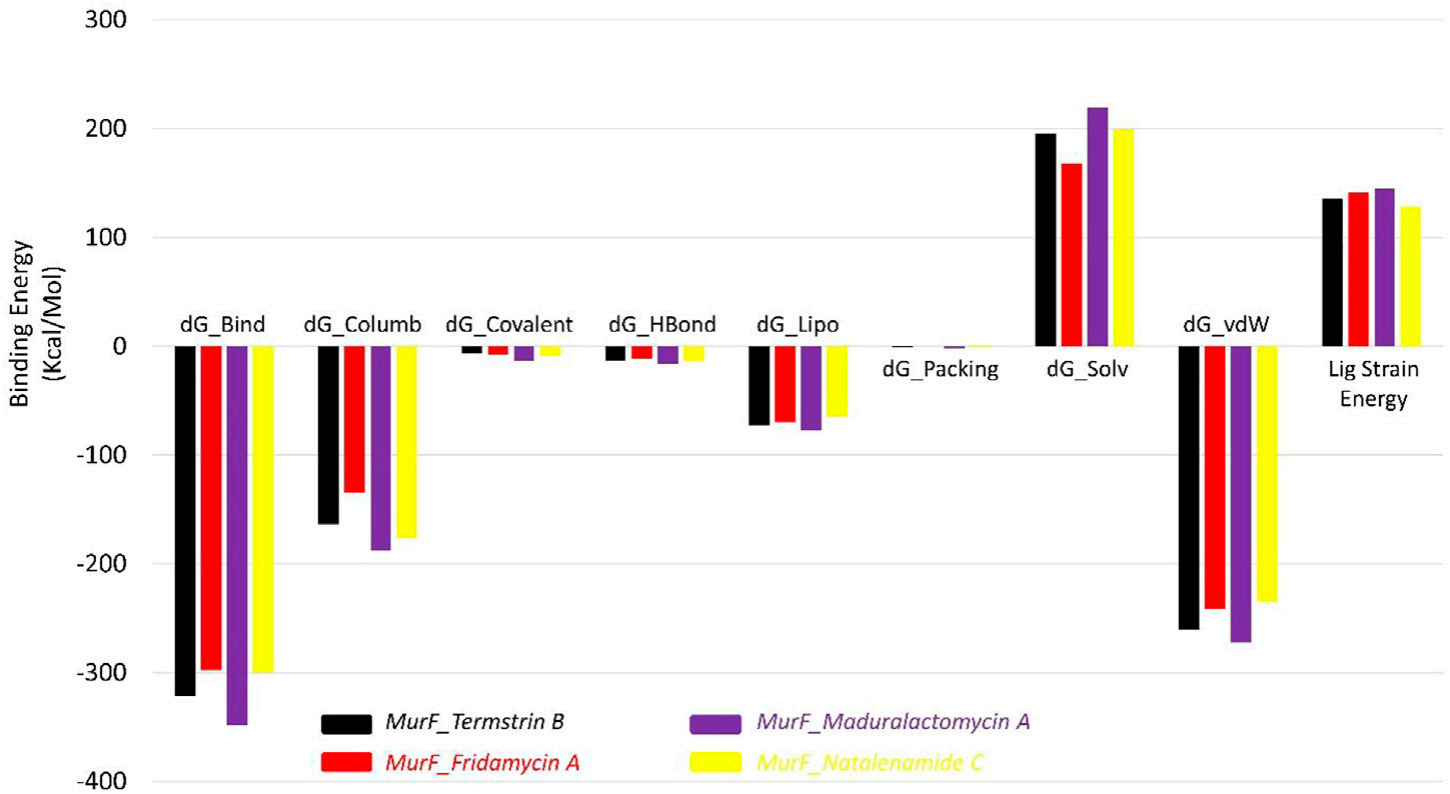
The Molecular mechanics, Generalized Born, and surface area solvation energies for Termstrin B (black), Fridamycin A (red), and Maduralactomycin A (indigo) and Natalenamide C (yellow) in complex with MurF (4ZIY) of *A. baumannii*.

The results of the MMGBSA analysis show that the compound Maduralactomycin A has the strongest binding energy among the five compounds, with a total energy of −348.48 kcal/mol. This is followed by Termstrin B at −321.19 kcal/mol, Natalenamide C at −299.65 kcal/mol, and Fridamycin A at −297.20 kcal/mol. This indicates that these compounds have relatively high binding affinities for the protein. The largest contributors to the binding energy are the van der Waals interactions and the solvation energy by the implicit solvent, with −260.18 kcal/mol and 195.57 kcal/mol, respectively. Electrostatic interactions also play a significant role in the binding energy, with −163.63 kcal/mol. For Maduralactomycin A, Natalenamide C and Fridamycin A are van der Waals interactions (−272.31 kcal/mol, −234.53 kcal/mol, and −241.08 kcal/mol) and solvation by implicit solvent (219.20 kcal/mol, 199.28 kcal/mol, and 167.48 kcal/mol). While the contributions from covalent, hydrogen bonding, lipophilic, and packing interactions are relatively small, they all play a role in the overall binding energy. The ligand strain energy is also provided, which is a measure of the energy required to deform the ligand into the binding pocket of the protein. The highest ligand strain energy is observed for Maduralactomycin A, at 144.9 kcal/mol (Figure 9). This suggests that this compound may have a more complex binding mode than the other compounds. Overall, the MMGBSA analysis provides a comprehensive understanding of the different types of interactions that contribute to the binding energy of the compounds to the protein. The relative energy values obtained from the simulation can help to rank the compounds according to their binding affinities for the protein and understand the underlying molecular interactions."

## Discussion

The increasing prevalence of MDR *A. baumannii* infections has become a major public health concern globally (Motbainor et al., 2020). *A. baumannii* is a Gram-negative bacterium that is one of the six most common causes of MDR hospital-acquired infections and is responsible for several severe infections such as pneumonia, bacteremia, and urinary tract infections (A Rahman et al., 2017; Lowe et al., 2018). The bacterium has intrinsic resistance to a broad range of antibiotics, and acquired resistance mechanisms, such as enzymes inactivating antibiotics, have emerged. The high resistance rate of *A. baumannii* to Ceftazidime (94.11%), (Cefepime 88.23%) imipenem (77.77%) (Abed et al., 2023) and high mortality rates (50%-64%) have made it a priority-I pathogen according to the World Health Organization, which placed critical category for the urgent need of new antibiotics (Kieny, 2017).

In recent years, the Mur family of enzymes has become a focus of antibacterial therapy research. The MurF enzyme is essential for the survival of bacteria and is extensively conserved in both Gram-positive and Gram-negative bacteria, making it a potential target for broad-spectrum drugs (Smith, 2006). Inhibitors of MurF have been proposed, including pseudo-tripeptides and sulfonamide inhibitors, thiazolyl aminopyrimidine compounds, 8-hydroxyquinolines, and 4-phenylpiperidine derivatives (Baum et al., 2006; Baum et al., 2007; Baum et al., 2009). Since new therapeutic development is challenging, time-consuming, and costly. Bioinformatics studies have been phenomenal in genome-wide studies (GWAS) to identify mutations (Khattak et al., 2021; Ahmad et al., 2022a), its annotation (Ahmad et al., 2022b; Shah et al., 2022) and impact (Ijaz et al., 2021; Ali et al., 2022a), identification of novel and potent drugs (Ajmal et al., 2022; Ali et al., 2023; Rehman et al., 2023), its targets (Jan et al., 2021) and candidate transformations (Zahid et al., 2023). *In silico* methods are significant to define the position of a gene, predict its transcripts, interaction with neighbors (Ali et al., 2022b), and determine the function and structure of a protein generated from that gene within the cell (Scott et al., 2008). *In silico* study also supports us to differentiate the neutral and deleterious SNPs through various algorithm and accessible information in the databases (Yue and Moult, 2006) and their role in the drug discovery. Increasing multidrug resistance against *P. aeruginosa* highlighted the need for effective treatments to combat this pathogen. The application of *in-silico*-based modelling techniques compensates for the inherent limitations of natural products and offers a unique opportunity to re-establish natural products as a major source for drug discovery (Lahlou, 2013; Sehgal et al., 2013; Sehgal et al., 2016). Studies using computational biology techniques, such as molecular modelling and docking, have shown the potential of various antibacterial agents to bind and inhibit key enzymes or receptors in bacteria (Sehgal et al., 2013; Sehgal et al., 2018).

Insect-associated bacteria have been recognized as a very promising natural source for discovering novel bioactive secondary metabolites (Beemelmanns et al., 2016; WycheTryptorubin et al., 2017). The high rediscovery rate of known natural products has diminished the enthusiasm to explore new natural products from historically important natural sources such as medicinal plants. Fungus-growing termite gut-derived natural inhibitors, such as alkaloids, PKS, and NRPS, have also been shown to potentially bind and inhibit microbial growth, making them attractive targets for screening potential antibacterial drugs (BeemelmannsMacrotermycins et al., 2017; Lee et al., 2018b; Liu et al., 2018; Yin et al., 2019; Zhang et al., 2020; Long et al., 2022; Zhou et al., 2022). The identification of new and effective antimicrobial agents against MDR A. baumannii is crucial for overcoming the public health threat posed by this bacterium. Using molecular docking and molecular dynamic simulation approaches this study demonstrated that four compounds, Termstrin B, Fridamycin A, Maduralactomycin A, and Natalenamide C, have the potential to be inhibitors of the MurF protein of *A. baumannii*. The Lipinski validation of the compounds indicates that all four compounds have no violations of Lipinski’s rules of five, meaning they are predicted to have good oral bioavailability. Table 1 summarizes the molecular properties of the four compounds, and Table 2 provides information on their predicted drug-like properties, including solubility, gastrointestinal absorption, bioavailability score, and synthetic accessibility.

Based on the results of the RMSD, RMSF, and β-factor analysis, Maduralactomycin A is found to be the most stable compound against the MurF of *A. baumannii*. The RMSD data shows that Maduralactomycin A has the lowest average RMSD value and the lowest standard deviation, suggesting a more stable interaction with the MurF protein compared to the other compounds. The RMSF analysis reveals that the residues Glu6, Pro7, and Trp8 are the most rigid, while the β-factor data also supports the regions of high flexibility identified by RMSF analysis. These regions may play a significant role in the binding and interaction of the compounds with the MurF protein. Furthermore, Maduralactomycin A was found to have the best overall performance in terms of RMSF values. These findings indicate that Maduralactomycin A has a more stable and consistent interaction with the MurF protein, making it a promising candidate for further drug development against *A. baumannii*. The hydrogen bond analysis provides information on the number of hydrogen bonds formed between the compounds and the MurF protein. The average number of hydrogen bonds formed varies among the compounds, with Termstrin B having the highest at 404.67, followed by Maduralactomycin A and Natalenamide C, both with an average of 393.67. Fridamycin A forms the least number of hydrogen bonds, with an average of 392.32. The standard deviation of the number of hydrogen bonds is also provided, with Fridamycin A having the highest at 9.44 and Termstrin B having the lowest at 5.36. The highest maximum number of inter-protein hydrogen bonds is observed for Termstrin B at 424 and the highest minimum number is observed for Natalenamide C at 365. These results suggest that the number of hydrogen bonds formed can provide insight into the strength of the interaction between the compound and the MurF protein. Termstrin B appears to form the strongest interaction with the protein, while Fridamycin A has the greatest variability in the number of hydrogen bonds formed, as indicated by its high standard deviation. These results can be used to further understand the molecular interactions and binding modes of the compounds with the MurF protein.

It is important to note that while the compounds show promise as inhibitors of the MurF protein, it is crucial to validate these results experimentally before reaching any conclusions. Some of the predicted properties, such as solubility and bioavailability score, can differ between the two logarithmic scales used in the analysis, highlighting the need for further experimental validation. Despite these limitations, the results of this study provide valuable information for the development of new inhibitors for *A. baumannii* and suggest that these four compounds especially Maduralactomycin A, and Natalenamide C warrant further investigation for their potential as treatments against this pathogen.

## Conclusion

The study aimed to evaluate the potential of 374 fungal-associated termite tripartite-derived natural products as inhibitors of the essential bacterial enzyme MurF of MDR *A. baumannii*. After molecular docking and computational pharmacokinetics screening, four compounds, Termstrin B, Fridamycin A, Maduralactomycin A, and Natalenamide C, were found to have higher binding affinities towards the MurF protein. The results showed that Maduralactomycin A was the most stable compound against MurF with the lowest average RMSD value and low standard deviation from RMSD analysis. Additionally, Maduralactomycin A was found to have the strongest binding energy among all the compounds and the most favourable dynamics. Termstrin B was also found to have a strong interaction with the protein and the highest average number of hydrogen bonds. These findings suggest that Maduralactomycin A and Termstrin B are potential candidates for further drug development against *A. baumannii*. However, experimental validation through animal models and clinical trials is recommended before reaching any final conclusions.

## Data Availability

The original contributions presented in the study are included in the article/Supplementary Material, further inquiries can be directed to the corresponding authors.

## References

[B1] A Rahman N. I. Ismail S. Alattraqchi A. G. Cleary D. W. Clarke S. C. (2017). Acinetobacter spp. infections in Malaysia: A review of antimicrobial resistance trends, mechanisms and epidemiology. Front. Microbiol. 8, 2479. 10.3389/fmicb.2017.02479 29312188 PMC5733036

[B2] Aanen D. K. Ros V. I. D. de Fine Licht H. H. Mitchell J. de Beer Z. W. Slippers B. (2007). Patterns of interaction specificity of fungus-growing termites and Termitomyces symbionts in South Africa. BMC Evol. Biol. 7 (1), 115–211. 10.1186/1471-2148-7-115 17629911 PMC1963455

[B3] Abed A. B. Korcan S. E. Güngör S. (2023). Antibiotics profile map of clinical A. Baumannii strains isolated from health institutions in Turkey: A database search study and analysis of publications from 2011 to 2022. Bull. Natl. Res. Centre 47 (1), 15. 10.1186/s42269-023-00982-6

[B4] Ahmad S. U. Ali Y. Jan Z. Rasheed S. Nazir N. U. A. Khan A. (2022). Computational screening and analysis of deleterious nsSNPs in human p14ARF (CDKN2A gene) protein using molecular dynamic simulation approach. J. Biomol. Struct. Dyn., 1–12. 10.1080/07391102.2022.2059570 35446184

[B5] Ahmad S. U. Hafeez Kiani B. Abrar M. Jan Z. Zafar I. Ali Y. (2022). A comprehensive genomic study, mutation screening, phylogenetic and statistical analysis of SARS-CoV-2 and its variant omicron among different countries. J. Infect. Public Health 15 (8), 878–891. 10.1016/j.jiph.2022.07.002 35839568 PMC9262654

[B6] Ajmal A. Ali Y. Khan A. Wadood A. Rehman A. U. (2022). Identification of novel peptide inhibitors for the KRas-G12C variant to prevent oncogenic signaling. J. Biomol. Struct. Dyn., 1–10. 10.1080/07391102.2022.2138550 36300526

[B7] Ali Y. Ahmad F. Ullah M. F. Haq N. U. Haq M. I. U. Aziz A. (2022). Structural evaluation and conformational dynamics of *ZNF141* ^T474I^ mutation provoking postaxial polydactyly type A. Bioengineering 9 (12), 749. 10.3390/bioengineering9120749 36550955 PMC9774408

[B8] Ali Y. Imtiaz H. Tahir M. M. Gul F. Saddozai U. A. K. Ur Rehman A. (2023). Fragment-based approaches identified tecovirimat-competitive novel drug candidate for targeting the F13 protein of the monkeypox virus. Viruses 15 (2), 570. 10.3390/v15020570 36851785 PMC9959752

[B9] Ali Y. Yasin M. Aziz A. Wajid Khan A. Rahman S. u. Ul Haq N. (2022). *In-silico* analysis of 2-cysteine peroxiredoxin genes in arabidopsis thaliana with possible role in carbon dioxide fixation through carbonic anhydrase regulation. Pak. J. Biochem. Biotechnol. 3 (1), 175–189. 10.52700/pjbb.v3i1.126

[B10] Banerjee P. Eckert A. O. Schrey A. K. Preissner R. (2018). ProTox-II: A webserver for the prediction of toxicity of chemicals. Nucleic acids Res. 46 (W1), W257–W263. 10.1093/nar/gky318 29718510 PMC6031011

[B11] Barreteau H. Kovac A. Boniface A. Sova M. Gobec S. Blanot D. (2008). Cytoplasmic steps of peptidoglycan biosynthesis. FEMS Microbiol. Rev. 32 (2), 168–207. 10.1111/j.1574-6976.2008.00104.x 18266853

[B12] Baum E. Z. Crespo-Carbone S. M. Abbanat D. Foleno B. Maden A. Goldschmidt R. (2006). Utility of muropeptide ligase for identification of inhibitors of the cell wall biosynthesis enzyme MurF. Antimicrob. agents Chemother. 50 (1), 230–236. 10.1128/AAC.50.1.230-236.2006 16377691 PMC1346814

[B13] Baum E. Z. Crespo-Carbone S. M. Foleno B. D. Simon L. D. Guillemont J. Macielag M. (2009). MurF inhibitors with antibacterial activity: Effect on muropeptide levels. Antimicrob. agents Chemother. 53 (8), 3240–3247. 10.1128/AAC.00166-09 19470511 PMC2715636

[B14] Baum E. Z. Crespo-Carbone S. M. Klinger A. Foleno B. D. Turchi I. Macielag M. (2007). A MurF inhibitor that disrupts cell wall biosynthesis in *Escherichia coli* . Antimicrob. agents Chemother. 51 (12), 4420–4426. 10.1128/AAC.00845-07 17908943 PMC2167990

[B15] Beemelmanns C. Guo H. Rischer M. Poulsen M. (2016). Natural products from microbes associated with insects. Beilstein J. Org. Chem. 12 (1), 314–327. 10.3762/bjoc.12.34 26977191 PMC4778507

[B16] BeemelmannsMacrotermycins C. A–D. Kim K. H. Klassen J. L. Cao S. Wyche T. P. (2017). Macrotermycins A-D, glycosylated macrolactams from a termite-associated amycolatopsis sp. M39. Org. Lett. 19 (5), 1000–1003. 10.1021/acs.orglett.6b03831 28207275 PMC6006516

[B17] Bowers K. J. (2006). “Scalable algorithms for molecular dynamics simulations on commodity clusters,” in Proceedings of the 2006 ACM/IEEE conference on supercomputing.

[B18] Brownlie J. C. Johnson K. N. (2009). Symbiont-mediated protection in insect hosts. Trends Microbiol. 17 (8), 348–354. 10.1016/j.tim.2009.05.005 19660955

[B19] Bugg T. D. Braddick D. Dowson C. G. Roper D. I. (2011). Bacterial cell wall assembly: Still an attractive antibacterial target. Trends Biotechnol. 29 (4), 167–173. 10.1016/j.tibtech.2010.12.006 21232809

[B20] Chem3D C. (1991). Scientific computing. Cambridge, Massachusetts: Cambridge Scientific Computing. Inc.

[B21] Chen T. Wang L. Li Q. Long Y. Lin Y. Yin J. (2020). Functional probiotics of lactic acid bacteria from Hu sheep milk. BMC Microbiol. 20 (1), 228–312. 10.1186/s12866-020-01920-6 32723292 PMC7390111

[B22] Cho G.-S. Rostalsky A. Fiedler G. Rösch N. Igbinosa E. (2018). Diversity and antibiotic susceptibility of Acinetobacter strains from milk powder produced in Germany. Front. Microbiol. 9, 536. 10.3389/fmicb.2018.00536 29636733 PMC5880893

[B23] Chouvenc T. Efstathion C. A. Elliott M. L. Su N. Y. (2013). Extended disease resistance emerging from the faecal nest of a subterranean termite. Proc. R. Soc. B Biol. Sci. 280 (1770), 20131885. 10.1098/rspb.2013.1885 PMC3779336 24048157

[B24] Comess K. M. Schurdak M. E. Voorbach M. J. Coen M. Trumbull J. D. Yang H. (2006). An ultraefficient affinity-based high-throughout screening process: Application to bacterial cell wall biosynthesis enzyme MurF. SLAS Discov. 11 (7), 743–754. 10.1177/1087057106289971 16973923

[B25] Correa A. Del Campo R. Escandón-Vargas K. Perenguez M. Rodríguez-Baños M. Hernández-Gómez C. (2018). Distinct genetic diversity of carbapenem-resistant Acinetobacter baumannii from Colombian hospitals. Microb. Drug Resist. 24 (1), 48–54. 10.1089/mdr.2016.0190 28570118 PMC5802270

[B26] Cousins K. R. (2005). ChemDraw ultra 9.0. CambridgeSoft, 100 CambridgePark drive, cambridge, MA 02140. Www. cambridgesoft.com. See web site for pricing options. J. Am. Chem. Soc. 127 (11), 4115–4116. 10.1021/ja0410237

[B27] Daina A. Michielin O. Zoete V. (2017). SwissADME: A free web tool to evaluate pharmacokinetics, drug-likeness and medicinal chemistry friendliness of small molecules. Sci. Rep. 7 (1), 42717. 10.1038/srep42717 28256516 PMC5335600

[B28] DeLano W. L. (2002). Pymol: An open-source molecular graphics tool. CCP4 Newsl. Protein Crystallogr. 40 (1), 82–92.

[B29] El Zoeiby A. Sanschagrin F. Levesque R. C. (2003). Structure and function of the mur enzymes: Development of novel inhibitors. Mol. Microbiol. 47 (1), 1–12. 10.1046/j.1365-2958.2003.03289.x 12492849

[B30] Ellwood M. D. Foster W. A. (2004). Doubling the estimate of invertebrate biomass in a rainforest canopy. Nature 429 (6991), 549–551. 10.1038/nature02560 15175749

[B31] Gu Y. G. Florjancic A. S. Clark R. F. Zhang T. Cooper C. S. Anderson D. D. (2004). Structure–activity relationships of novel potent MurF inhibitors. Bioorg. Med. Chem. Lett. 14 (1), 267–270. 10.1016/j.bmcl.2003.09.073 14684340

[B32] Guo H. Schwitalla J. W. Benndorf R. Baunach M. Steinbeck C. Görls H. (2020). Gene cluster activation in a bacterial symbiont leads to halogenated angucyclic maduralactomycins and spirocyclic actinospirols. Org. Lett. 22 (7), 2634–2638. 10.1021/acs.orglett.0c00601 32193935

[B33] Halgren T. A. (1996). Merck molecular force field. I. Basis, form, scope, parameterization, and performance of MMFF94. J. Comput. Chem. 17 (5‐6), 490–519. 10.1002/(sici)1096-987x(199604)17:5/6<490:aid-jcc1>3.0.co;2-p

[B34] Hanwell M. D. Curtis D. E. Lonie D. C. Vandermeersch T. Zurek E. Hutchison G. R. (2012). Avogadro: An advanced semantic chemical editor, visualization, and analysis platform. J. Cheminformatics 4 (1), 17. 10.1186/1758-2946-4-17 PMC3542060 22889332

[B35] Hrast M. Anderluh M. Knez D. Randall C. P. Barreteau H. O'Neill A. J. (2014). Design, synthesis and evaluation of second generation MurF inhibitors based on a cyanothiophene scaffold. Eur. J. Med. Chem. 73, 83–96. 10.1016/j.ejmech.2013.11.031 24384549

[B36] Ijaz A. Shah K. Aziz A. Rehman F. U. Ali Y. Tareen A. M. (2021). Novel frameshift mutations in XPC gene underlie xeroderma pigmentosum in Pakistani families. Indian J. Dermatol 66 (2), 220–222. 10.4103/ijd.IJD_63_20 34188291 PMC8208272

[B37] Ingti B. Upadhyay S. Hazarika M. Khyriem A. B. Paul D. Bhattacharya P. (2020). Distribution of carbapenem resistant Acinetobacter baumannii with blaADC-30 and induction of ADC-30 in response to beta-lactam antibiotics. Res. Microbiol. 171 (3-4), 128–133. 10.1016/j.resmic.2020.01.002 31988011

[B38] Jacobson M. P. Friesner R. A. Xiang Z. Honig B. (2002). On the role of the crystal environment in determining protein side-chain conformations. J. Mol. Biol. 320 (3), 597–608. 10.1016/s0022-2836(02)00470-9 12096912

[B39] Jan Z. Ahmad S. U. Qadus A. Ali Y. Sajjad W. Rais F. (2021). Insilico structural and functional assessment of hypothetical protein L345_13461 from Ophiophagus hannah. Pure Appl. Biol. 10 (4), 1109–1118. 10.19045/bspab.2021.100116

[B40] Jones G. Willett P. Glen R. C. Leach A. R. Taylor R. (1997). Development and validation of a genetic algorithm for flexible docking. J. Mol. Biol. 267 (3), 727–748. 10.1006/jmbi.1996.0897 9126849

[B41] Jorgensen W. L. Chandrasekhar J. Madura J. D. Impey R. W. Klein M. L. (1983). Comparison of simple potential functions for simulating liquid water. J. Chem. Phys. 79 (2), 926–935. 10.1063/1.445869

[B42] Jorgensen W. L. Maxwell D. S. Tirado-Rives J. (1996). Development and testing of the OPLS all-atom force field on conformational energetics and properties of organic liquids. J. Am. Chem. Soc. 118 (45), 11225–11236. 10.1021/ja9621760

[B43] Khattak S. Rauf M. A. Zaman Q. Ali Y. Fatima S. Muhammad P. (2021). Genome-wide analysis of codon usage patterns of SARS-CoV-2 virus reveals global heterogeneity of COVID-19. Biomolecules 11 (6), 912. 10.3390/biom11060912 34207362 PMC8233742

[B44] Khurshid M. Rasool M. H. Ashfaq U. A. Aslam B. Waseem M. Xu Q. (2020). Dissemination of blaOXA-23-harbouring carbapenem-resistant Acinetobacter baumannii clones in Pakistan. J. Glob. Antimicrob. Resist. 21, 357–362. 10.1016/j.jgar.2020.01.001 32006748

[B45] Kieny M.-P. (2017). WHO publishes list of bacteria for which new antibiotics are urgently needed. 27 February 2017 [cited 2023 03 March]; Available from: https://www.who.int/news/item/27-02-2017-who-publishes-list-of-bacteria-for-which-new-antibiotics-are-urgently-needed .

[B46] Kim S. Chen J. Cheng T. Gindulyte A. He J. He S. (2021). PubChem in 2021: New data content and improved web interfaces. Nucleic Acids Res. 49 (D1), D1388–d1395. 10.1093/nar/gkaa971 33151290 PMC7778930

[B47] Kräutler V. Van Gunsteren W. F. Hünenberger P. H. (2001). A fast SHAKE algorithm to solve distance constraint equations for small molecules in molecular dynamics simulations. J. Comput. Chem. 22 (5), 501–508. 10.1002/1096-987x(20010415)22:5<501:aid-jcc1021>3.0.co;2-v

[B48] Kroiss J. Kaltenpoth M. Schneider B. Schwinger M. G. Hertweck C. Maddula R. K. (2010). Symbiotic streptomycetes provide antibiotic combination prophylaxis for wasp offspring. Nat. Chem. Biol. 6 (4), 261–263. 10.1038/nchembio.331 20190763

[B49] Lahlou M. (2013). The success of natural products in drug discovery Pharmacol. Pharmacy.

[B50] Lee S. Goo J. Y. (2004). The PreADME: Pc-based program for batch prediction of adme properties. EuroQSAR 9, 5–10.

[B51] Lee S. R. Natalenamides A–C. Yu J. S. Benndorf R. Lee D. S. (2018). Natalenamides A⁻C, cyclic tripeptides from the termite-associated *actinomadura* sp. RB99. Molecules 23 (11), 3003. 10.3390/molecules23113003 30453579 PMC6278286

[B52] Lee S. R. Song J. H. Song J. H. Ko H. J. Baek J. Y. Trinh T. A. (2018). Chemical identification of isoflavonoids from a termite-associated Streptomyces sp. RB1 and their neuroprotective effects in murine hippocampal HT22 cell line. Int. J. Mol. Sci. 19 (9), 2640. 10.3390/ijms19092640 30200599 PMC6164413

[B53] Lipinski C. A. Lombardo F. Dominy B. W. Feeney P. J. (1997). Experimental and computational approaches to estimate solubility and permeability in drug discovery and development settings. Adv. drug Deliv. Rev. 23 (1-3), 3–26. 10.1016/s0169-409x(00)00129-0 11259830

[B54] Liu C. Chang Y. Xu Y. Luo Y. Wu L. Mei Z. (2018). Distribution of virulence-associated genes and antimicrobial susceptibility in clinical Acinetobacter baumannii isolates. Oncotarget 9 (31), 21663–21673. 10.18632/oncotarget.24651 29774093 PMC5955172

[B55] Long Y. Zhang Y. Huang F. Liu S. Gao T. Zhang Y. (2022). Diversity and antimicrobial activities of culturable actinomycetes from Odontotermes formosanus (Blattaria: Termitidae). BMC Microbiol. 22 (1), 80. 10.1186/s12866-022-02501-5 35337263 PMC8951712

[B56] Lowe M. Ehlers M. M. Ismail F. Peirano G. Becker P. J. Pitout J. D. D. (2018). Acinetobacter baumannii: Epidemiological and beta-lactamase data from two tertiary academic hospitals in tshwane, south Africa. Front. Microbiol. 9, 1280. 10.3389/fmicb.2018.01280 29946315 PMC6005857

[B57] Lu C. Wu C. Ghoreishi D. Chen W. Wang L. Damm W. (2021). OPLS4: Improving force field accuracy on challenging regimes of chemical space. J. Chem. Theory Comput. 17 (7), 4291–4300. 10.1021/acs.jctc.1c00302 34096718

[B58] Ma Y. X. Wang C. Y. Li Y. Y. Li J. Wan Q. Q. Chen J. H. (2020). Considerations and caveats in combating ESKAPE pathogens against nosocomial infections. Adv. Sci. 7 (1), 1901872. 10.1002/advs.201901872 PMC6947519 31921562

[B59] Manjula A. Pushpanathan M. Sathyavathi S. Gunasekaran P. Rajendhran J. (2016). Comparative analysis of microbial diversity in termite gut and termite nest using ion sequencing. Curr. Microbiol. 72, 267–275. 10.1007/s00284-015-0947-y 26613615

[B60] Mohammed Ali H. S. Altayb H. N. Bayoumi A. A. M. El Omri A. Firoz A. Chaieb K. (2022). *In silico* screening of the effectiveness of natural compounds from algae as SARS-CoV-2 inhibitors: Molecular docking, ADMT profile and molecular dynamic studies. J. Biomol. Struct. Dyn. 41, 3129–3144. 10.1080/07391102.2022.2046640 35253618

[B61] Molinspiration Log P. (2017). Calculation of molecular properties and bioactivity score.

[B62] Motbainor H. Bereded F. Mulu W. (2020). Multi-drug resistance of blood stream, urinary tract and surgical site nosocomial infections of acinetobacter baumannii and *Pseudomonas aeruginosa* among patients hospitalized at felegehiwot referral hospital, northwest Ethiopia: A cross-sectional study. BMC Infect. Dis. 20 (1), 92–11. 10.1186/s12879-020-4811-8 32000693 PMC6993407

[B63] Moubareck C. A. Halat D. H. (2020). *Insights into Acinetobacter baumannii: A Review of microbiological.* Virulence, and resistance traits in a threatening nosocomial pathogen. 10.3390/antibiotics9030119 PMC7148516 32178356

[B64] Otani S. Zhukova M. Koné N. A. da Costa R. R. Mikaelyan A. Sapountzis P. (2019). Gut microbial compositions mirror caste‐specific diets in a major lineage of social insects. Environ. Microbiol. Rep. 11 (2), 196–205. 10.1111/1758-2229.12728 30556304 PMC6850719

[B65] Poulsen M. (2015). Towards an integrated understanding of the consequences of fungus domestication on the fungus‐growing termite gut microbiota. Environ. Microbiol. 17 (8), 2562–2572. 10.1111/1462-2920.12765 25581852

[B66] Presta L. Bosi E. Mansouri L. Dijkshoorn L. Fani R. Fondi M. (2017). Constraint-based modeling identifies new putative targets to fight colistin-resistant A. baumannii infections. Sci. Rep. 7 (1), 3706–3712. 10.1038/s41598-017-03416-2 28623298 PMC5473915

[B67] Rashid M. Afzal O. Altamimi A. S. A. (2021). Benzimidazole molecule hybrid with oxadiazole ring as antiproliferative agents: *In-silico* analysis, synthesis and biological evaluation. J. Chil. Chem. Soc. 66 (2), 5164–5182. 10.4067/s0717-97072021000205164

[B68] Rehman A. U. Khurshid B. Ali Y. Rasheed S. Wadood A. Ng H. L. (2023). Computational approaches for the design of modulators targeting protein-protein interactions. Expert Opin. Drug Discov. 18 (3), 315–333. 10.1080/17460441.2023.2171396 36715303 PMC10149343

[B69] Rosengaus R. B. Traniello J. F. Bulmer M. S. (2010). “Ecology, behavior and evolution of disease resistance in termites,” in Biology of termites: A modern synthesis (Springer), 165–191.

[B70] Santajit S. Indrawattana N. (2016). Mechanisms of antimicrobial resistance in ESKAPE pathogens. BioMed Res. Int. 2016, 2475067. 10.1155/2016/2475067 27274985 PMC4871955

[B71] Saxena S. Abdullah M. Sriram D. Guruprasad L. (2018). Discovery of novel inhibitors of *Mycobacterium tuberculosis* MurG: Homology modelling, structure based pharmacophore, molecular docking, and molecular dynamics simulations. J. Biomol. Struct. Dyn. 36 (12), 3184–3198. 10.1080/07391102.2017.1384398 28948866

[B72] Schneider T. Sahl H.-G. (2010). An oldie but a goodie–cell wall biosynthesis as antibiotic target pathway. Int. J. Med. Microbiol. 300 (2-3), 161–169. 10.1016/j.ijmm.2009.10.005 20005776

[B73] Scott J. J. Oh D. C. Yuceer M. C. Klepzig K. D. Clardy J. Currie C. R. (2008). Bacterial protection of beetle-fungus mutualism. Science 322 (5898), 63. 10.1126/science.1160423 18832638 PMC2761720

[B74] Sehgal S. A. Hammad M. A. Tahir R. A. Akram H. N. Ahmad F. (2018). Current therapeutic molecules and targets in neurodegenerative diseases based on *in silico* drug design. Curr. Neuropharmacol. 16 (6), 649–663. 10.2174/1570159X16666180315142137 29542412 PMC6080102

[B75] Sehgal S. A. Khattak N. A. Mir A. (2013). Structural, phylogenetic and docking studies of D-amino acid oxidase activator (DAOA), a candidate schizophrenia gene. Theor. Biol. Med. Model. 10 (1), 3. 10.1186/1742-4682-10-3 23286827 PMC3553032

[B76] Sehgal S. A. Mannan S. Ali S. (2016). Pharmacoinformatic and molecular docking studies reveal potential novel antidepressants against neurodegenerative disorders by targeting HSPB8. Drug Des. Dev. Ther. 10, 1605–1618. 10.2147/DDDT.S101929 PMC4866741 27226709

[B77] Seipke R. F. Barke J. Brearley C. Hill L. Yu D. W. Goss R. J. M. (2011). A single Streptomyces symbiont makes multiple antifungals to support the fungus farming ant Acromyrmex octospinosus. PLoS one 6 (8), e22028. 10.1371/journal.pone.0022028 21857911 PMC3153929

[B78] Shah A. A. Amjad M. Hassan J. U. Ullah A. Mahmood A. Deng H. (2022). Molecular insights into the role of pathogenic nsSNPs in GRIN2B gene provoking neurodevelopmental disorders. Genes 13 (8), 1332. 10.3390/genes13081332 35893069 PMC9394290

[B79] Shahzad S. Willcox M. Shahzad A. (2020). Identification of novel *in vitro* antibacterial action of cloprostenol and evaluation of other non-antibiotics against multi-drug resistant A. baumannii. J. Antibiotics 73 (1), 72–75. 10.1038/s41429-019-0244-2 31586155

[B80] Shaik M. S. Liem S. Y. Yuan Y. Popelier P. L. A. (2010). Simulation of liquid imidazole using a high-rank quantum topological electrostatic potential. Phys. Chem. Chem. Phys. 12 (45), 15040–15055. 10.1039/c0cp00417k 20967311

[B81] Skariyachan S. Manjunath M. Bachappanavar N. (2019). Screening of potential lead molecules against prioritised targets of multi-drug-resistant-Acinetobacter baumannii–insights from molecular docking, molecular dynamic simulations and *in vitro* assays. J. Biomol. Struct. Dyn. 37 (5), 1146–1169. 10.1080/07391102.2018.1451387 29529934

[B82] Smith C. A. (2006). Structure, function and dynamics in the mur family of bacterial cell wall ligases. J. Mol. Biol. 362 (4), 640–655. 10.1016/j.jmb.2006.07.066 16934839

[B83] Sobral R. Ludovice A. M. Gardete S. Tabei K. De Lencastre H. Tomasz A. (2003). Normally functioning murF is essential for the optimal expression of methicillin resistance in *Staphylococcus aureus* . Microb. Drug Resist. 9 (3), 231–241. 10.1089/107662903322286436 12959401

[B84] Sujada N. Sungthong R. Lumyong S. (2014). Termite nests as an abundant source of cultivable actinobacteria for biotechnological purposes. Microbes Environ. 29 (2), 211–219. 10.1264/jsme2.me13183 24909709 PMC4103528

[B85] Turk S. Kovac A. Boniface A. Bostock J. M. Chopra I. Blanot D. (2009). Discovery of new inhibitors of the bacterial peptidoglycan biosynthesis enzymes MurD and MurF by structure-based virtual screening. Bioorg. Med. Chem. 17 (5), 1884–1889. 10.1016/j.bmc.2009.01.052 19223185

[B86] Verma P. Tiwari M. Tiwari V. (2018). *In silico* high-throughput virtual screening and molecular dynamics simulation study to identify inhibitor for AdeABC efflux pump of Acinetobacter baumannii. J. Biomol. Struct. Dyn. 36 (5), 1182–1194. 10.1080/07391102.2017.1317025 28393677

[B87] WycheTryptorubin T. P. A. Schwab L. Currie C. R. Clardy J. (2017). Tryptorubin A: A polycyclic peptide from a fungus-derived streptomycete. J. Am. Chem. Soc. 139 (37), 12899–12902. 10.1021/jacs.7b06176 28853867 PMC5609116

[B88] Yin C. Jin L. Li S. Xu X. Zhang Y. (2019). Diversity and antagonistic potential of Actinobacteria from the fungus-growing termite Odontotermes formosanus. 3 Biotech. 9, 45–47. 10.1007/s13205-019-1573-3 PMC6342738 30729069

[B89] Yoon S.-Y. Lee S. R. Hwang J. Y. Benndorf R. Beemelmanns C. Chung S. J. (2019). Fridamycin A, a microbial natural product, stimulates glucose uptake without inducing adipogenesis. Nutrients 11 (4), 765. 10.3390/nu11040765 30939853 PMC6520714

[B90] Yue P. Moult J. (2006). Identification and analysis of deleterious human SNPs. J. Mol. Biol. 356 (5), 1263–1274. 10.1016/j.jmb.2005.12.025 16412461

[B91] Zahid S. Ali Y. Rashid S. (2023). Structural-based design of HD-TAC7 PROteolysis TArgeting chimeras (PROTACs) candidate transformations to abrogate SARS-CoV-2 infection. J. Biomol. Struct. Dyn., 1–16. 10.1080/07391102.2023.2183037 36841549

[B92] Zhang L. Song T. Wu J. Zhang S. Yin C. Huang F. (2020). Antibacterial and cytotoxic metabolites of termite-associated Streptomyces sp. BYF63. J. Antibiotics 73 (11), 766–771. 10.1038/s41429-020-0334-1 32533072

[B93] Zhang Y.-l. Li S. Jiang D. h. Kong L. c. Zhang P. h. Xu J. d. (2013). Antifungal activities of metabolites produced by a termite-associated Streptomyces canus BYB02. J. Agric. food Chem. 61 (7), 1521–1524. 10.1021/jf305210u 23360202

[B94] Zhang Y. L. Ge H. M. Li F. Song Y. C. Tan R. X. (2008). New phytotoxic metabolites from Pestalotiopsis sp. HC02, a fungus residing in Chondracris rosee gut. Chem. Biodivers. 5 (11), 2402–2407. 10.1002/cbdv.200890204 19035568

[B95] Zhou L. Wang J. Wu F. Yin C. Kim K. H. Zhang Y. (2022). Termite nest associated Bacillus siamensis YC-9 mediated biocontrol of Fusarium oxysporum f. sp. cucumerinum. Front. Microbiol. 13, 893393. 10.3389/fmicb.2022.893393 35722323 PMC9198579

